# Update on Non-Alcoholic Fatty Liver Disease-Associated Single Nucleotide Polymorphisms and Their Involvement in Liver Steatosis, Inflammation, and Fibrosis: A Narrative Review

**DOI:** 10.52547/ibj.3647

**Published:** 2022-08-22

**Authors:** Fajar Dwi Astarini, Neneng Ratnasari, Widya Wasityastuti

**Affiliations:** 1 Master in Biomedical Sciences, Faculty of Medicine, Public Health and Nursing, Universitas Gadjah Mada, 55281 Indonesia;; 2 Subdivision of Gastroenterohepatology, Department of Internal Medicine, Dr. Sardjito Hospital, Faculty of Medicine, Public Health and Nursing, Universitas Gadjah Mada, Yogyakarta, 55281 Indonesia;; 3 Department of Physiology, Faculty of Medicine, Public Health and Nursing, Universitas Gadjah Mada, Yogyakarta, 55281 Indonesia

**Keywords:** Fibrosis, Inflammation, Non-alcoholic fatty liver disease, Polymorphism

## Abstract

Genetic factors are involved in the development, progression, and severity of NAFLD. Polymorphisms in genes regulating liver functions may increase liver susceptibility to NAFLD. Therefore, we conducted this literature study to present recent findings on NAFLD-associated polymorphisms from published articles in PubMed from 2016 to 2021. From 69 selected research articles, 20 genes and 34 SNPs were reported to be associated with NAFLD. These mutated genes affect NAFLD by promoting liver steatosis (PNPLA3, MBOAT7, TM2SF6, PTPRD, FNDC5, IL-1B, PPARGC1A, UCP2, TCF7L2, SAMM50, IL-6, AGTR1, and NNMT), inflammation (PNPLA3, TNF-α, AGTR1, IL-17A, IL-1B, PTPRD, and GATAD2A), and fibrosis (IL-1B, PNPLA3, MBOAT7, TCF7L2, GATAD2A, IL-6, NNMT, UCP, AGTR1, and TM2SF6). The identification of these genetic factors helps to better understand the pathogenesis pathways of NAFLD

## INTRODUCTION

Non-alcoholic fatty liver disease is a term commonly used to cover an array of clinical manifestations in the liver that are not induced by secondary causes such as alcohol or drug consumption and defined genetic disorders. These manifestations involve steatosis, inflammation, and fibrosis, which can lead to cirrhosis and even hepatocellular carcinoma^[^^1^^-^^3^^]^. Histologically, NAFLD is classified into NAFL and NASH. In NAFL, steatosis is seen in more than 5% of the parenchyma, while in NASH, necroinflammatory is present alongside steatosis. Obesity and insulin resistance drive the accumulation of TGs and FFAs in the liver, contributing to the growing epidemic of NAFLD^[^^2^^]^. The average global prevalence rate of NAFLD is 25.24%, with the highest rates reported in the Middle East and South American countries reaching up to 30%. In Asia, the incidence of NAFLD is 50.9 cases per 1,000 person-years. The global prevalence of NAFLD has increased from 15% in 2005 to 25% in 2010, and it keeps increasing steadily^[4]^. More noticeable growth in NAFLD prevalence has been observed in Asia and Pacific countries, which might be correlated with the increasing rate of obesity, type 2 diabetes, and metabolic syndromes in this region^[^^4^^,^^5^^]^. 

It has been established that genetic factors, along with environmental factors, are involved in the development, progression, and severity of NAFLD^[6]^. Certain genetic variants confer susceptibility to NAFLD. Several SNPs have been reported to be associated with specific phenotypes of NAFLD. Identifying the genetic factors in NAFLD will help to better understand the pathogenesis pathways of the disease. It also serves as a potential solution for future NAFLD genetic screening, the development of new genetic-based treatments, as well as the development of genetically modified animal models to facilitate studies in the field^[^^6^^]^. 

Similar reviews have previously been conducted on NAFLD-associated polymorphisms. Duvnjak *et al.*^[^^7^^]^ reviewed the genetic polymorphisms in NAFLD published between 2002 and 2009 and discussed their involvement in NAFLD development and progression. Severson *et al.*^[^^6^^]^ also reported the genetic factors affecting NAFLD from studies between 2012 and 2016, emphasizing certain genes and polymorphisms. A more recent article from Trépo and Valenti^[^^8^^] ^has reviewed several selected gene polymorphisms and their implications for NAFLD pathobiology, drug discovery, and risk prediction. In this narrative review, we aimed to present recent findings on NAFLD-associated polymorphisms from published articles in PubMed from 2016 to 2021 and focused on discussing their roles in three main NAFLD spectrums: steatosis, inflammation, and fibrosis.

## MATERIALS AND METHODS

**Fig. 1:**
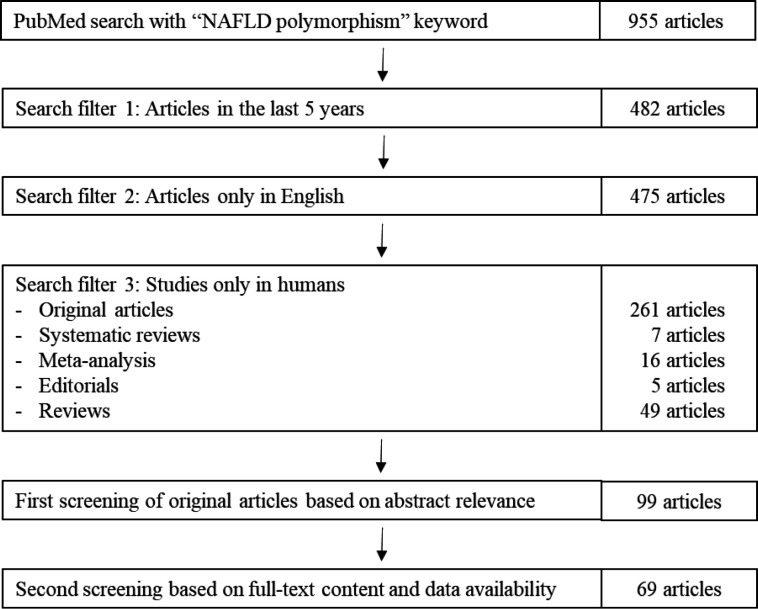
Article selection process.

We conducted a search in PubMed to identify the relevant articles. The detailed selection process is shown in Figure 1. The search term used was “NAFLD polymorphism” with the following search filters: published in the last five years (2016-2021), only in humans, and only articles in English. The search yielded 338 published references, which were then sorted by authors for relevance. Review articles and editorials were excluded from this study. Relevant research articles without complete data were also excluded. In the end, 69 published references were selected for this study, and the summarized data are presented in Table 1.

**Table 1:** NAFLD-associated SNPs published between 2016 and 2021

**Genes**	**SNP ID**	**Risk allele**	**Associations with NAFLD**
*PNPLA3*			
	*rs738409*	G	Aggravate hepatosteatosis^[9-17]^
			Development of NAFLD^[14,18-34]^
			Elevated alanine aminotransferase levels^[10,17,35-38]^
			Associated with NASH^[22,39-43]^
			Associated with hepatic fat fractions^[44]^
			Associated with hepatocyte ballooning^[41]^
			Lobular and portal inflammation^[41]^
			Increased liver graft fat content^[45]^
			Elevated level of TGs^[21,37]^
			Increased liver fibrosis^[13,14,17,22,34,36,42,46-50]^
			Associated with cirrhosis^[22]^
			Increased AST levels^[13,34,37,38]^
			Higher body mass index^[37]^
			Higher serum level of γ-glutamyltransferase, ALP, total cholesterol, LDL, and uric acid^ [37]^
			Higher serum level of CK18-M30^[14]^
			Increased severity of liver histology^[33,49]^
			Increased steatohepatitis, low level of high-density lipoprotein, and higher insulin resistance^[17]^
		
	*rs4823173*	A	Associated with increased AST levels^[51]^
		
	*rs2896019*	G	Associated with increased AST levels^[51,52]^
			Associated with NAFLD^[52,53]^
			Associated with increased ALT levels and decreased serum TGs and higher levels of LDL^[52]^

		
	*rs2281135*	A	Associated with AST levels^[51]^
			Associated with hepatocyte ballooning and NASH^[41]^
			Lobular and portal inflammation^[41]^
			Associated with NAFLD^[27,54]^
			Associated with advanced fibrosis^[50]^
		
	*rs3810622*	T	Associated with NAFLD, increased ALT levels, and higher level of blood glucose^[52]^
			Elevated ALT levels^[35]^
		
	*rs12483959*	A	Associated with NAFLD^[27]^
	*rs143392071*	G	Increased NAFLD risk^[55]^
	*rs2143571*	A	Associated with advanced fibrosis^[50]^
*MBOAT7*			
	*rs626283*	C	Associated with NAFLD and may affect glucose metabolism by modulating intrahepatic fat content^[56]^
	*rs641738*	T	Contributes to hepatic inflammation^[57]^
			Increased fibrosis^[13,57,58]^
			Higher ALT levels^[58,59]^
			Associated with increased liver injury^[13]^
			Associated with NAFLD risk^[14,24]^
			Associated with severe hepatic steatosis^[14,58]^
*TM6SF2*			
	*rs58542926*	T	Associated with or independent risk factors of hepatic steatosis^[13,60,61]^
			Elevated ALT levels^[13,61]^
			Independent predictors of NASH^[60]^
			Increased levels of aminotransferases^[36]^
			Associated with advanced fibrosis^[32]^
			Associated with the risk of NAFLD^[23,24,37,61,62]^
			Associated with liver injury, deleterious effects on liver health, modulate hepatic fat accumulation, and Increased serum AST^[13]^
			
*IL-17A*	*rs2275913*	A	Development of NAFLD in obese patients^[63^^]^
*COL13A1*	*rs1227756*	A	Higher risk of elevated ALT levels^[35]^
*SAMM50*	*rs3761472*	G	Associated with hepatocyte ballooning, lobular and portal inflammation, and NASH^[41]^	
			Significant associations with NAFLD^[27]^	
	*rs2143571*	A	Significant associations with NAFLD^[27]^	
	*rs2073080*	T	Significant associations with NAFLD^[27]^	
*IL-6*	*rs1800795*	C	Associated with the development of NASH^[64]^	
			Higher risk of steatosis with less parenchymal damage^[65]^	
			Increased risk of NAFLD, higher BMI, fat mass, % body fat, waist circumference, serum TGs, total cholesterol, ALP, AST, and fasting insulin levels^[66]^	
	*rs10499563*	C	Associated with the presence of definitive NASH, increased ballooning, and Mallory bodies^[65]^	
*IL-1B*	*rs1143634*	T	Associated with advanced fibrosis and increased Mallory bodies^[65]^	
*FNDC5*	*rs3480*	G	More severe steatosis^[67]^	
*AGTR1*	*rs5186*	C	Predictor of NAFLD incidence and severity^[68]^	
*PPARGC1A*	*rs8192678*	A	Risk factor for the development of NAFLD^[69]^	
*CD82*	*rs2303861*	G	Involved in the development and progression of NAFLD^[70]^	
*UCP2*	*rs659366*	A	Higher risk of NAFLD^[71]^	
		T	Determinant of fibrosis severity^[15]^	
*TNF-α*	*rs1800629*	A	Higher risk of NASH development^[72]^	
	*rs1799964*	C	Independent risk factors contributing to histological progression of NASH^[73]^	
*NNMT*	*rs694539*	A	Risk factor for developing NAFLD and NASH, correlated with the steatosis degree^[74]^	
*HSD11B1*	*rs2235543* *rs12565406* *rs4844880*	C G T	Increased risk of NAFLD development and higher liver fat content^[75]^	
*PTPRD*	*rs35929428*	A	Associated with the development of NAFLD, play a role in hepatic lipid accumulation and fibrosis^[76]^	
*GATAD2A*	*rs4808199*	A	Associated with NAFLD^[53]^	
*TCF7L-2*	*rs7903146*	T	Independently associated with NAFLD^[77]^	
*TLL1*	*rs17047200*	T	Higher risk of advanced fibrosis^[46]^	

BMI, body mass index; ALP, alkaline phosphatase; LDL, low-density lipoprotein

## RESULTS AND DISCUSSION

**Fig. 2:**
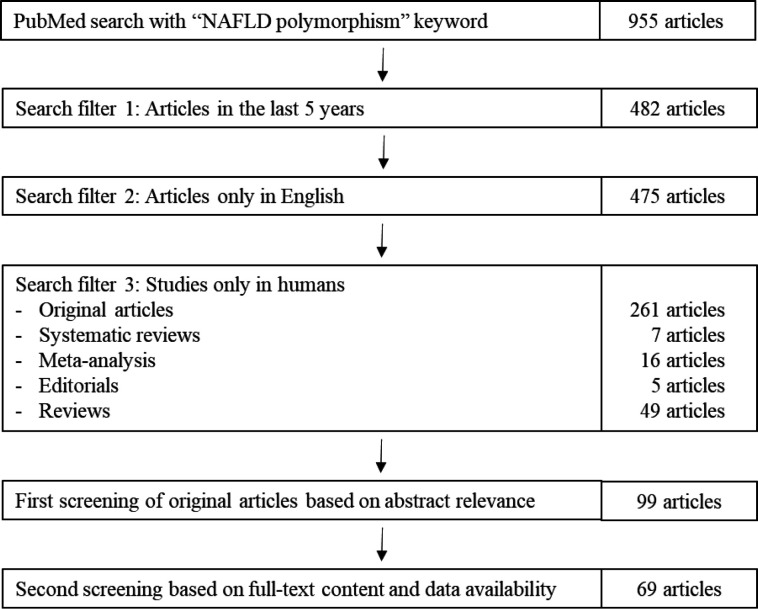
NAFLD-associated SNPs involved in liver steatosis. Polymorphisms in NAFLD-related genes cause TG accumulation in the liver through impaired TG hydrolase activities, increased lipogenesis, increased TG synthesis, reduced secretion of TG-rich VLDL, increased lipid droplet formation, STAT3 inactivation, increased liver supply of FFAs, decreased fatty acid oxidation, and decreased irisin secretion. Each gene and its polymorphisms have specific pathways in causing TG accumulation. For instance, polymorphisms in the *PNPLA3* gene can impair the TG hydrolase activities, as well as cause an increase in n-6 PUFA level, which stimulate lipogenesis in the liver, resulting in steatosis. Mutations in *TCF7L2*, *SAMM50*, *IL-6*, and *AGTR1* promote lipolysis in adipose tissue and skeletal muscle, leading to increased supply of FFAs to the liver, increased *de novo* lipogenesis, and eventually increased TG accumulation. Changes in *MBOAT7* and *IL-1B* genes cause increased TG synthesis and lipid droplet formation. Meanwhile, the *UCP2* gene seems to possess protective effects against steatosis by inducing fatty acid oxidation, lowering the supply of FFAs to the liver. (→: promote; **─|**: inhibit; : mutated genes; ↓: decreased; ↑: increased)

**Fig. 3:**
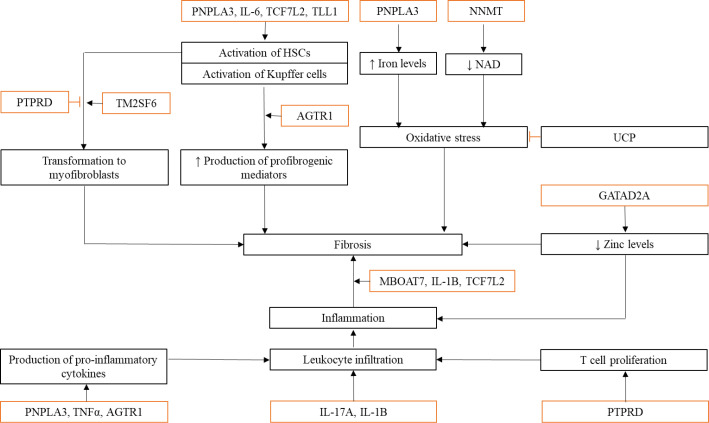
NAFLD-associated SNPs involved in liver inflammation and fibrosis. Inflammation is a contributing factor in fibrogenesis. Changes in genes involved in both processes can affect the development and progression of NAFLD. Mutations in *PTPRD*, *PNPLA3*, *TNF-α*, *AGTR1*, *IL-17A*, *IL-1B*, and *GATAD2A* indirectly cause fibrosis by inducing inflammatory responses through increased production of pro-inflammatory cytokines, increased immune cell proliferation, and leukocyte recruitment. Other polymorphisms are involved in fibrogenesis by either activating HSCs and Kupffer cells or inducing oxidative stress in liver tissue. Activated HSCs can transform into myofibroblasts that will then produce excess collagen, resulting in tissue scarring. (→: promote; **─|**: inhibit;  : mutated genes; ↓: decreased; ↑: increased)

GWAS has contributed to the identification of potential SNPs in NAFLD. These studies provided insights into the pathogenesis and the long-term prognosis of NAFLD^[^^78^^]^. There were 20 genes and 34 SNPs reported to be associated with NAFLD in studies published in the last five years, which matched our search parameters as presented in Table 1. The majority of the literature we used in this review has investigated the association of NAFLD with three arguably major genetic factors of NAFLD: *PNPLA3* *rs738409*, *TM6SF2* *rs58542926*, and *MBOAT7* *rs641738*. Each SNP has its roles in the development and progression of NAFLD, with the most reported association including the independent risk of NAFLD, aggravated steatosis, increased liver fibrosis, as well as elevated ALT and AST levels. Even though the association of the SNPs and NAFLD has been established in those genetic studies presented in Table 1, the involvement of each polymorphism in NAFLD is often unclear. In this review, we discuss the possible involvement of the genes and/or the variants in three NAFLD spectrums (steatosis, inflammation, and fibrosis) based on the published studies. We also drafted the possible relationships of the discussed genes in those NAFLD spectrums as shown in Figures 2 and 3. 

***PNPLA3***

The Human *PNPLA3* gene is located on chromosome 22, encoding a protein called adiponutrin. The gene acts as a lipid droplet regulator in hepatocytes, HSCs, and adipocytes. Since 1998, the *rs738409* C>G variant has been identified to be associated with NAFLD^[^^79^^,^^80^^]^. The variant was reported to be involved in hepatic steatosis, inflammation, and fibrosis. It is unclear how the variant affects liver TG content, but it has been demonstrated that the variant is associated with the loss of TG hydrolase activities, eventually increasing intrahepatic TG accumulation^[^^81^^]^. Accordingly, the variant was linked to higher levels of circulating TGs, corroborating the impaired TG hydrolysis by lipoprotein lipase^[21]^. The hepatic fat content in individuals carrying the variant has also shown an increase in n-6 polyunsaturated fatty acids, indicating a pro-inflammatory condition that promotes *de novo* lipogenesis in the liver^[^^81^^]^. *rs738409* was the only variant of *PNPLA3* associated with hepatic steatosis in this review. The *rs738409* SNP, as well as *rs2281135* and *rs2143571*, are also involved in hepatic fibrosis. *PNPLA3* has been reported to activate HSCs and promote migration, proliferation, and the pro-fibrogenic activities of HSCs^[^^82^^]^. Patients with NAFLD carrying the G allele of the *rs738409* variant have displayed elevated serum ferritin levels, as well. Iron can cause oxidative stress by interacting with oxygen radicals. Oxidative stress is implicated in mediating the progression of fibrosis. Iron can also induce fibrosis by activating Kupffer cells to release pro-fibrogenic mediators^[83]^. Chatterjee *et al.*^[^^41^^]^ have reported the association of *PNPLA3* variants, *rs738409* and *rs2281135*, with portal and lobular inflammation. The variants are correlated with the release of pro-inflammatory and pro-fibrogenic cytokines such as chemokine ligand 5, monocyte chemoattractant protein-1, IL-8, granulocyte-macrophage colony-stimulating factor, and TNF-α^[81]^. Individuals harbouring the *rs738409* variant had greater inflammatory infiltration than individuals with wild- type genotypes^[^^84^^]^. Accordingly, the culture medium of cells expressing the genetic variants was also shown to recruit more immune cells than the wild-type carriers^[^^81^^]^. To summarize, the polymorphisms in *PNPLA3* gene affect NAFLD development and progression by promoting steatosis (*rs738409*), inflammation (*rs738409 *and* rs2281135*), and fibrosis (*rs738409*,* rs2281135*, and* rs2143571*). However, there was limited information on the involvement of the other* PNPLA3* variants (*rs4823173, rs2896019, rs3810622*,* rs12483959*, and* rs143392071*) reported in this review on those three NAFLD spectrums.

***MBOAT7***

MBOAT7 protein, so-called lysophosphatidylinositol acyltransferase 1, is involved in acyl remodelling of phosphatidylinositols in the Lands cycle^[^^58^^]^. The carriers of *rs641738* T allele have indicated lower hepatic *MBOAT7* mRNA and protein expression^[^^57^^]^. Lower *MBOAT7* expression is correlated with severe hepatic inflammation, advanced fibrosis, and higher ALT levels^[^^57^^-^^59^^]^. However, *MBOAT7* involvement in hepatic inflammation is still unclear. It has previously demonstrated that the strong expression of *MBOAT7* is found in immune cell subsets such as neutrophils, peripheral blood mononuclear cells, lymphocytes (B and T), monocytes, macrophages, natural killer cells, and dendritic cells^[^^85^^]^. The protein is also involved in eicosanoid production by neutrophils and myeloid cells, as well as the stimulation of T lymphocyte proliferation^[^^57^^]^. These findings suggest that *MBOAT7* plays a role in inflammatory activities. Also, *MBOAT7*-mediated inflammation is thought to be associated with the progression to fibrosis, possibly independent of lipid accumulation and insulin resistance as the *rs641738* variant was not associated with steatosis in chronic hepatitis B and C patients, as well as in obese Taiwanese children^[^^10^^,^^57^^,^^85^^]^. However, other studies have reported that the variant is also associated with steatosis^[14,58]^. In cultured human hepatocytes, reduced *MBOAT7* expression caused by the *rs641738* variant resulted in higher phosphatidylinositols turnover. This condition leads to the constant production of diacylglycerol, resulting in increased synthesis of hepatocyte TG^[^^86^^]^. TGs are known to be the main form of lipid stored in hepatic steatosis. In diet-induced steatotic mice, inhibition of TG synthesis through diacylglycerol acyltransferase 2-knockout could lower hepatic TGs by ~70%, with no significant changes in liver inflammation, fibrosis, and insulin-glucose metabolism^[^^87^^]^. This finding supports the hypothesis that inflammation and fibrosis caused by the *rs641738* variant are independent of lipid accumulation. Another variant of the *MBOAT7* gene, *rs626283*, has been exhibited to be related to liver fat content and impaired insulin sensitivity in obese Caucasian youth but not in African American and Hispanic populations^[^^56^^]^. Buch *et al.*^[^^88^^]^ have denoted that *rs626283* had the strongest association with severe liver damage at the *MBOAT7* locus in European descent individuals with alcohol-related cirrhosis. The variant was also in high linkage disequilibrium with the *rs641738* variant, the functional variant affecting *MBOAT7* expression^[^^88^^]^. However, the involvement of the *rs626283 *variant in NAFLD remains unclear. Taken together, both MBOAT7 variants contribute to NAFLD through inflammation-mediated fibrosis and steatosis. 

***TM6SF2***

The *TM6SF2* *rs58542926* SNP was identified to be associated with NAFLD, hepatic steatosis, elevated ALT and AST levels, and advanced fibrosis. A meta-analysis by Liu *et al.*^[^^89^^]^ has also pointed out that the *rs58542926* variant is associated with fibrosis and steatosis in individuals with chronic hepatitis C. Interestingly, the variant was not linked to inflammation. *rs58542926* is known to decrease TM6SF2 expression. The variant causes reduced secretion of TG-rich VLDL, leading to lower serum TG levels and increased intrahepatic TG accumulation^[6,89]^. It is unclear whether the fibrosis is driven by lipid accumulation or not. An *in vitro* study has demonstrated that the *rs58542926* SNP might increase the sensitivity of HSC activation. In the liver, HSCs are activated by TGF-β1, which is secreted by HSCs or Kupffer cells. TGF-β1 stimulates HSC transformation into myofibroblasts. *TM6SF2*-knockdown LX2 cells have shown increased mRNA expression of αsmooth muscle actin following TGF-β1 treatment, indicating that the variant promotes fibrosis^[^^90^^]^. The role of *TM6SF2* in fibrosis still requires further investigations. Altogether, *TM6SF2* *rs58542926* SNP is involved in NAFLD pathogenesis by promoting steatosis and fibrosis.

***PPARGC1A***

The A allele of *rs8192678* SNP in *PPARGC1A* gene is a risk factor for NAFLD development in adult Iranian and Chinese Han populations^[^^69^^,^^91^^]^. However, the SNP was not associated with the biochemical and physiological parameters investigated in the study, including body mass index, fasting blood sugar, creatine, TGs, plasma lipid levels, HbA1c, and microalbumin levels. *PPARGC1A* is a transcriptional factor involved in lipid and energy metabolism^[^^69^^]^. The gene encodes peroxisome proliferator-activated receptor PGC-1α, which is highly expressed in the liver. PGC-1α promotes fatty acid oxidation in fasting condition^[^^91^^]^. Liang and Ward^[^^92^^]^ have reported that the downregulation of this gene increased lipogenesis and steatosis in the liver. Accordingly, the *rs8192678* A allele was found to significantly lower the expression of *PPARGC1A*, resulting in reduced PGC-1α activities and altered PGC-1α interactions in regulating oxidative stress and lipid metabolism which will eventually lead to NAFLD development^[^^69^^,^^91^^]^. Overall, the *rs8192678* A allele contributes to NAFLD development through steatosis induction.

***IL-17A***

*IL-17*, especially *IL-17A*, is involved in NAFLD pathogenesis^[^^93^^]^. *IL-17* induces the production of IL-6, which is important for Th17 cell differentiation. The *rs2275913* (A) allele polymorphism is associated with elevated *IL-17A* levels^[^^94^^]^. Overexpression of *IL-17A* resulted in NAFLD progression and worsened liver injury in obese mice^[^^92^^]^. The IL-17A/IL-17RA axis is important in the progression of NAFL to NASH in high fat and methionine choline-deficient diets. Massive infiltration of IL-17^+^ cells was also found in NASH liver^[^^95^^]^. In conclusion, IL-17A SNP contributes to NAFLD development through its role in inflammation.

***IL-6***

Upregulation of serum and hepatic *IL-6* was observed in patients with NAFLD and animal models. In the liver, IL-6 is produced by hepatocytes and Kupffer cells, and its expression in hepatocytes is correlated with the disease severity. IL-6 has protective roles in the liver due to its antiapoptotic action and its involvement in improving hepatic regeneration and repair. However, prolonged overexpression of IL-6 might increase liver susceptibility to injury and apoptosis. IL-6 is recently known to be a mediator of fibrogenesis in HSCs. IL-6 also promotes the release of FFAs from the adipose tissue, increasing the supply of FFAs to the liver^[^^96^^]^. Both *rs1800795* (C) and *rs10499563* (C) alleles are polymorphisms in the promoter region of the *IL-6* gene. The former polymorphism is frequently associated with lower *IL-6* expression even though there were reports of its association with higher serum IL-6 levels^[^^66^^,^^97^^,^^98^^]^. Further studies are required to confirm the effects of these polymorphisms on IL-6 levels. Mutations in the *IL-6* gene weaken its hepatoprotective effect, making the liver more susceptible to NAFLD through inflammation, steatosis, and fibrosis.

***IL-1B***

*IL-1B* is involved in NAFLD development through the IL-1 receptor signaling pathway. The *rs1143634* polymorphism in *IL-1B* gene is suggested to be associated with higher *IL-1B* expression. The presence of IL-1B induces lipid droplet formation in hepatocytes. IL-1B also promotes the recruitment of neutrophils in the liver by upregulating the expression of intercellular adhesion molecule 1 in endothelial cells. IL-1B, IL-6, and TNF-α cause chronic inflammation in the liver by activating local immune cells and attracting other immune cells to the liver. IL-1B also contributes to the progression from liver inflammation to liver fibrosis^[^^99^^]^. *IL-1B* involvement in steatosis, inflammation, and fibrosis contributes to NAFLD development and progression. 

***TNF-α***

*TNF-α* is involved in the development and progression of NAFLD by inducing the production of lipid metabolism enzymes, proinflammatory cytokines, and fibrosis-associated proteins^[^^100^^]^. It activates proinflammatory pathways such as c-Jun N-terminal kinase and nuclear factor-κB and indirectly blocks the anti-inflammatory effect of insulin by contributing to the development of insulin resistance^[^^101^^,^^102^^]^. Studies have reported the overexpression of circulating *TNF-α* among patients with NAFLD. Both *rs1800629* A allele and *rs1799964* C allele are associated with higher *TNF-α* expression. The increased circulating *TNF-α* is correlated with NAFLD severity^[^^103^^]^. As a result, the SNPs in the *TNF-α *gene facilitate the progression to NASH through its role in inflammation, steatosis, and fibrosis.

***FNDC5***

Metwally *et al.*^[^^67^^]^ have reported an association between the *FNDC5* *rs3480* variant and advanced steatosis. The variant affects hepatic *FNDC5* expression and provides a binding site for miR-135a-5P that regulates several pathways involved in liver disease. *FNDC5* is known to secrete irisin, which can ameliorate steatosis. A study by Canivet *et al.*^[^^104^^]^ have shown that *FNDC5* could prevent fat accumulation in hepatocytes *in vitro*^. ^The genetic variant was found to downregulate *FNDC5* expression^[^^67^^]^. Therefore, the lower expression of *FNDC5* due to the polymorphism can lead to more severe steatosis. This observation suggests that without the polymorphism, liver tissue would express higher *FNDC5* for its protective properties^[^^104^^]^. In summary, the *FNDC5* variant is involved in NAFLD by causing advanced steatosis.

***COL13A1***

Larrieta-Carrasco *et al.*^[^^35^^]^ have reported that the carriers of *rs1227756* variant in *COL13A1* gene expressed elevated ALT and AST levels, even though only the elevated AST level was significantly associated with *rs1227756*. However, the mechanism underlying the condition is still unclear. The variant was also reported to be associated with lobular inflammation in patients with NAFLD and T2DM^[^^105^^]^. Increased aminotransferase levels often indicate the presence of inflammation^[^^106^^]^. It is possible that changes in *COL13A1* gene may influence the levels of liver enzymes through inflammatory response and/or T2DM-related pathways. Further studies are required to elucidate the involvement of *COL13A1* in elevating the transaminase levels. To summarize, *COL13A1 *polymorphism may contribute to NAFLD through inflammation.

***CD82***

A variant of *CD82* was found to be associated with the development and progression of NAFLD. The mechanism by which the *rs2303861* polymorphism influences NAFLD pathophysiology is still unclear due to the limited availability of studies on the topic. It is theorized that the polymorphism in *CD82* gene promotes hepatic steatosis based on the evidence that *CD82*-knockout mice exhibit increased adipogenic potential. The *rs2303861* SNP is also in linkage disequilibrium with *rs7942159* of the *PNPLA2* gene, which is involved in fat mobilization in adipose tissue^[^^70^^]^. Further studies are needed to investigate the effects of *CD82* on NAFLD. However, it is thought that the CD82 variant plays a role in the development and progression of NAFLD through steatosis. 

***AGTR1***

The *AGTR1* *rs5186* C allele can predict the risk and severity of NAFLD in Caucasian and Iranian populations^[^^68^^,^^107^^]^. The polymorphism promotes fat-induced proinflammatory response and enhances NF-κB activation in mononuclear cells. Activated NF-κB induces the release of pro-inflammatory and pro-fibrogenic adipokines and chemokines, resulting in inflammation, adipose tissue dysfunction, and hepatic injury in NASH. The C allele of the polymorphism causes insulin resistance in skeletal muscle and adipose tissue, increasing the supply of FFAs to the liver and the release of mainly pro-inflammatory adipokines and chemokines^[^^68^^,^^108^^]^. The C allele is also responsible for VLDL accumulation, which is rich in TGs and cholesterol^[68]^. Collectively, the *AGTR1* *rs5186* C allele can be a predictor of NAFLD incidence and severity due to its involvement in inflammation, steatosis, and fibrosis in NAFLD.

***UCP2***

The *rs659366* G>A and C>T of the *UCP2* gene are correlated with NAFLD susceptibility and fibrosis severity, respectively^[^^15^^,^^71^^]^. The carriers of *rs659366* A allele are at higher risk of developing NAFLD in Iranian population with NAFLD^[^^71^^]^. The AA genotype shows the high expression of *UCP2* and oxidative stress markers, as well as reduced insulin production^[^^109^^]^. However, the involvement of *UCP2* in the development and progression of NAFLD is still unclear. Theoretically, *UCP2* may have protective activities against NAFLD. High plasma fatty acid supply in the liver induces higher expression of UCP2. UCP2 will then promote fatty acid oxidation through several mechanisms: (1) increasing beta-oxidation of fatty acid in the mitochondria, (2) translocating non-esterified fatty acids to prevent accumulation in the mitochondrial matrix, (3) releasing FFAs from the mitochondrial matrix and allowing re-entry as acyl-CoA required for beta-oxidation, and (4) activating AMP-activated protein kinase, promoting the use of fatty acids in energy metabolism. Nevertheless, it has not been proven that *UCP2* can prevent steatosis. Controversy also arises over the involvement of *UCP2* in oxidative stress. *UCP2* is thought to be able to prevent ROS formation, but there is not enough evidence to support this claim. Increased *UCP2* expression is still unable to reduce oxidative stress and ROS formation in NAFLD animal models^[110]^. Ultimately, polymorphisms in the *UCP2* gene may disrupt its protective roles in the liver and contribute to NAFLD development through steatosis and fibrosis.

***TCF7L-2***

The *rs7903146* T allele in the *TCF7L-2* gene was found to be strongly associated with NAFLD in Asian Indian population^[^^77^^]^. The T allele of this polymorphism is correlated with the increased expression of *TCF7L-2*^[^^111^^]^. TCF7L-2 modulates the activation of HSCs and fibrogenesis in the liver through β-catenin/TCF pathway. *TCF7L-2* is also expressed in adipose tissue. *TCF7L-2* activation in adipose tissue leads to inflammation, lipolysis, and lower adiponectin production^[^^112^^]^. Increased lipolysis following *TCF7L-2* activation results in higher serum FFAs. Reduced adiponectin production will disrupt glucose and fatty acid metabolism. In line with these findings, the *rs7903146* polymorphism was reported to be associated with a high level of serum FFAs^[^^113^^]^. TCF7L-2 also regulates glucose homeostasis, the *rs7903146* variant impaired insulin secretion, making the carriers of the polymorphism at risk of developing T2DM^[^^77^^]^. This condition causes insulin resistance that contributes to the development of NAFLD by increasing *de novo* lipogenesis in the liver and promoting lipolysis in other tissues, leading to a higher FFA supply to the liver^[^^114^^]^. In short, the *rs7903146 *variant is involved in the development and progression of NAFLD through steatosis, inflammation, and fibrosis.

***SAMM50***

The *rs738491* T allele, *rs2143571* A allele, and *rs3761472* G allele of *SAMM50* gene are associated with NAFLD in Korean population^[^^27^^]^. The association of *SAMM50* polymorphisms and NAFLD has also been reported in Japanese, Asian Indian (only *rs3761472 *SNP), and Chinese Han populations^[^^41^^,^^115^^,^^116^^]^. The *rs3761472* variant was reported to be associated with hepatocyte ballooning, lobular and portal inflammation, and NASH^[^^41^^]^. The gene itself,* SAMM50*, plays a role in the progression of NAFL to NASH. *SAMM50* encodes Sam50, which is important in maintaining the structure of mitochondrial cristae and the assembly of mitochondrial respiratory chain complexes^[^^116^^]^. Downregulation of Sam50 in the liver can cause mitochondrial dysfunction, which is known to contribute to the development of insulin resistance and hepatic steatosis in obese rat model^[^^117^^]^. Liver biopsy from patients with NASH has also shown mitochondrial abnormalities^[118]^. The three *SAMM50* variants may cause the lower production of Sam50, leading to mitochondrial dysfunction-mediated steatosis. To sum up, those variants may be responsible for NAFLD development and progression through inflammation (*rs3761472 *only), steatosis, and insulin resistance. 

***TLL1***

The *rs17047200* T allele is associated with a higher risk of developing advanced fibrosis in Japanese patients with NAFLD^[46]^. The SNP leads to the elevated expression of *TLL1*, which has been found to activate HSCs in animal models and humans, indicating its involvement in fibrogenesis^[^^119^^].^ Activated HSCs have a myofibroblast-like phenotype, contributing to fibrogenesis through cell proliferation and upregulation of matrix production^[^^120^^]^. However, a contradicting study by Bayoumi *et al.*^[^^121^^]^ has reported that *rs17047200* is not associated with fibrosis in Caucasian patients with biopsy-proven metabolic-associated fatty liver disease. That study has also demonstrated that the overexpression of *TLL1* in HSCs is detected in patients with metabolic steatohepatitis, in a murine fibrosis model fed with methionine choline-deficient diet and in an *in vitro* human fibrosis model. Further studies are needed to elucidate the roles of *TLL1* in both steatohepatitis and fibrosis, as well as to confirm the effects of *rs17047200* SNP on NAFLD. However, it is theorized that the SNP affects NAFLD through its involvement in steatosis and fibrosis. 

***NNMT***

The AA genotype of *NNMT* *rs694539* variant is related to the increased risk of NASH in obese Egyptians. The SNP is associated with steatosis but it is not considered a fibrosis marker^[^^74^^].^ Komatsu *et al.*^[^^122^^]^ have reported that the overexpression of *NNMT* depleted nicotinamide adenine dinucleotide (NAD) and *S*-adenosylmethionine, inducing the genes involved in steatosis and fibrosis in the liver of transgenic mice overexpressing *NNMT*. NAD has protective effects on ROS and also facilitates hydrogen transfer in reductive or oxidative metabolic reactions. NAD depletion reduces fatty acid oxidation, leading to the accumulation of TGs in hepatocytes. Therefore, inhibiting *NNMT* activities may prevent progression to NASH. Briefly, changes in the *NNMT* gene may contribute to NAFLD through steatosis and ROS-mediated fibrosis.

***HSD11B1***

The *rs2235543*, *rs12565406*, and *rs4844880* polymorphisms in *HSD11B1* gene are associated with the liver fat content. Accordingly, the *HSD11B1* mRNA expression positively correlates with the liver fat content, suggesting the involvement of 11β-HSD1 in hepatic fat accumulation. The homozygous major allele carriers of the three SNPs also have shown elevated expression of *HSD11B1* gene, and they are also twice at risk of developing NAFLD^[^^75^^]^. Overexpression of 11β-HSD1 in a high-fat diet leads to steatosis, while its deficiency is protective against steatosis. Lower expression of 11β-HSD1 is observed in the early stages of NAFLD, but increased 11β-HSD1 levels are required for the progression to NASH. Inhibition of 11β-HSD1 caused reduced lipid content, making it a potential therapeutic target for steatosis^[123]^. To summarize, the *HSD11B1* variants contribute to NAFLD through steatosis. 

***PTPRD***

Polymorphism in *PTPRD* gene may be related to hepatic lipid accumulation and fibrosis progression in Japanese patients with NAFLD. More advanced steatosis and fibrosis have been observed in the GA genotype of the *rs35929428 *variant. *PTPRD* mainly dephosphorylates STAT3. Based on this evidence, Nakajima *et al.*^[^^76^^]^ observed an association between *rs35929428* SNP and STAT3 dephosphorylation and found that the SNP enhanced STAT3 dephosphorylation and strongly suppressed its phosphorylation in hepatocytes. However, dephosphorylation is known to negatively regulate STAT3 activation^[^^124^^]^. STAT3 inactivation leads to TG accumulation and worsens steatosis and hepatocellular damage. STAT3 inactivation also inhibits fibroblast-to-myofibroblast transition in cultured fibroblasts, preventing the development of fibrosis^[^^125^^]^, which is in contrast to the results of Nakajima *et al.*’s^[^^76^^]^ study. The rs35929428 polymorphism may exacerbate fibrosis through other signalling pathways. In conclusion, the *PTPRD* variant has a role in the development and progression of NAFLD through steatosis and fibrosis. 

***GATAD2A***

*GATAD2A* has been reported to be associated with the increased risk of NAFLD in Japanese patients with NAFLD. However, the function of this gene in NAFLD development is still vague^[^^53^^,^^126^^]^. *GATAD2A* gene is located at 19p12, along with *TM6SF2* and *NCAN*, which are known to be associated with NAFLD^[126]^. *GATAD2A* enables zinc ion binding. In NASH, the serum zinc level is lower than in normal condition^[^^53^^]^. Zinc deficiency is a common pathogenesis pathway of NAFLD. Low zinc level correlates with more severe fibrosis and lobular nflammation^[^^126^^]^. The *rs4808199* polymorphism might cause the overexpression of *GATAD2A*, resulting in higher zinc ion binding and lower zinc serum level. Taken together, the variant might be involved in NAFLD pathogenesis through zinc-related inflammation and fibrosis. 

**Conflicting findings on NAFLD-associated polymorphisms and study limitations**

While the presented NAFLD-associated SNPs harbor potential benefits as therapeutic targets, conflicting results arise from several studies. For instance, no association was observed between NAFLD and the *rs58542926* variant of *TM6SF2* gene in Brazilian patients with NAFLD^[^^127^^]^. *MBOAT7* *rs641738* variant also did not show any correlation with steatosis in chronic hepatitis B and C patients, as well as in obese Taiwanese children^[^^10^^,^^57^^,^^85^^]^. These results might be due to the limitations of GWAS. In conventional GWAS, the association is only significant when it reaches the *p* < 5 × 10^-8 ^threshold. Owing to this high level of significance, the association might be undetected in studies with small sample sizes. The use of larger sample size is preferable even though it is not always possible to assemble a large sample size. Besides, GWAS cannot identify the causal variants and genes^[^^128^^]^. Therefore, further investigation of the SNPs and genes of interest *in vitro* and *in vivo* is important to fully understand their involvement in the development and progression of the disease. 

NAFLD is a complex disease. Many factors are involved in its development and progression. This review only presented the association of single genetic variants with NAFLD. NAFLD is known to be multigenic, involving the synergistic and antagonistic actions of several genes, along with environmental factors. Providing the information on combined NAFLD-associated SNPs would be a point of interest for future studies. We also could only infer the possible involvement of the SNPs and genes with their associated features in NAFLD from published literature, further studies are required to investigate the nature of their association.

## CONCLUSION

Genetic factors are involved in the development and progression of NAFLD. Identifying genetic factors in NAFLD help to better understand the pathogenesis pathways of the disease. The SNPs presented in this review affect NAFLD through their involvement in three NAFLD spectrums (steatosis, fibrosis, and inflammation). Mutations in PNPLA3, MBOAT7, TM2SF6, PTPRD, FNDC5, IL-1B, PPARGC1A, UCP2, and NNMT directly induce steatosis in the liver, while polymorphisms in *TCF7L2*, *SAMM50*, *IL-6*, and *AGTR1* genes indirectly promote liver steatosis by increasing lipolysis in adipose tissue and skeletal muscle, resulting in a higher supply of FFAs to the liver. SNPs in PNPLA3, TNF-α, AGTR1, IL-17A, IL-1B, PTPRD, and GATAD2A cause liver inflammation. Inflammation, along with mutations in IL-1B, PNPLA3, MBOAT7, TCF7L2, GATAD2A, IL-6, NNMT, UCP, AGTR1, and TM2SF6 also contributes to liver fibrosis. On the contrary, polymorphism in the *PTPRD* gene can inhibit fibrosis by preventing the transformation of HSCs to myofibroblasts. Even though these NAFLD-associated SNPs show potential benefits as therapeutic targets, conflicting findings from similar studies arise due to the limitations of GWAS. Therefore, further investigation of those genes and SNPs *in vitro* and *in vivo* is important to fully understand their involvement in the development and progression of NAFLD.

## DECLARATIONS

### Acknowledgments

The authors would like to thank the Ministry of Research and Technology, the Republic of Indonesia for the financial support by providing the Penelitian Dasar Unggulan Perguruan Tinggi (PDUPT) grant (reference number: 1674/UN1/DITLIT/DIT-LIT/PT/2021 and 1642/UN1/DITLIT/DIT-LIT/PT.01.03/2022).

### Ethical statement

Not applicable.

### Data availability

All data presented in the manuscript can be accessed on PubMed.

### Author contributions

FDA, NR, and WW contributed equally to the study conception, design, material preparation, data collection, data analysis, manuscript drafting, and manuscript writing. All authors have read and approved the final manuscript.

### Conflict of interest

None declared.

### Funding/support

This study has been supported by Penelitian Dasar Unggulan Perguruan Tinggi (PDUPT) grant (reference number: 1674/UN1/DITLIT/DIT-LIT/PT/2021 and 1642/UN1/DITLIT/DIT-LIT/PT.01.03/2022) from the Ministry of Research and Technology, Republic of Indonesia.
